# Magnetic Resonance Guided Radiotherapy for Head and Neck Cancers

**DOI:** 10.3390/jcm11051388

**Published:** 2022-03-03

**Authors:** Laila A. Gharzai, Benjamin S. Rosen, Bharat Mittal, Michelle L. Mierzwa, Poonam Yadav

**Affiliations:** 1 Department of Radiation Oncology, Northwestern University, Chicago, IL 60611, USA; laila.gharzai@northwestern.edu (L.A.G.); bharat.mittal@nm.org (B.M.); 2 Department of Radiation Oncology, University of Michigan, Ann Arbor, MI 48109, USA; rosenbs@med.umich.edu (B.S.R.); mmierzwa@med.umich.edu (M.L.M.)

**Keywords:** radiotherapy, magnetic resonance imaging, head and neck cancer, MR-Linac

## Abstract

Radiotherapy is an integral component of head/neck squamous cell carcinomas (HNSCCs) treatment, and technological developments including advances in image-guided radiotherapy over the past decades have offered improvements in the technical treatment of these cancers. Integration of magnetic resonance imaging (MRI) into image guidance through the development of MR-guided radiotherapy (MRgRT) offers further potential for refinement of the techniques by which HNSCCs are treated. This article provides an overview of the literature supporting the current use of MRgRT for HNSCC, challenges with its use, and developing research areas.

## 1. Introduction

The delivery of radiotherapy (RT) for cancer treatment was revolutionized in the 1990s with the development of computed tomography (CT)-based three-dimensional RT planning and image-guided RT (IGRT). This allowed for better targeting of tumors and areas at risk while sparing nearby normal tissues. Early approaches to IGRT replaced the utilization of external skin markings and included first fluoroscopy then later portal imaging, including kilovoltage and megavoltage imaging, which had limited soft tissue delineation but allowed for anatomic targeting.

The development of cone beam computed tomography (CBCT) around 2000 ushered in an era of further precision in RT, allowing for dose escalation aiming to eradicate tumors while sparing nearby tissues. Refinement of IGRT techniques has allowed for the proliferation of advanced radiotherapy techniques. Treatment of head and neck squamous cell carcinomas (HNSCCs) in particular has benefited from improvements in technology. Modern approaches to RT for HNSCC include salivary-sparing [1,2] and pharyngeal constrictor-sparing [3,4] approaches, which require more conformal dose distributions made possible by the improvement in technology used to delivery radiotherapy.

**Figure 1:**
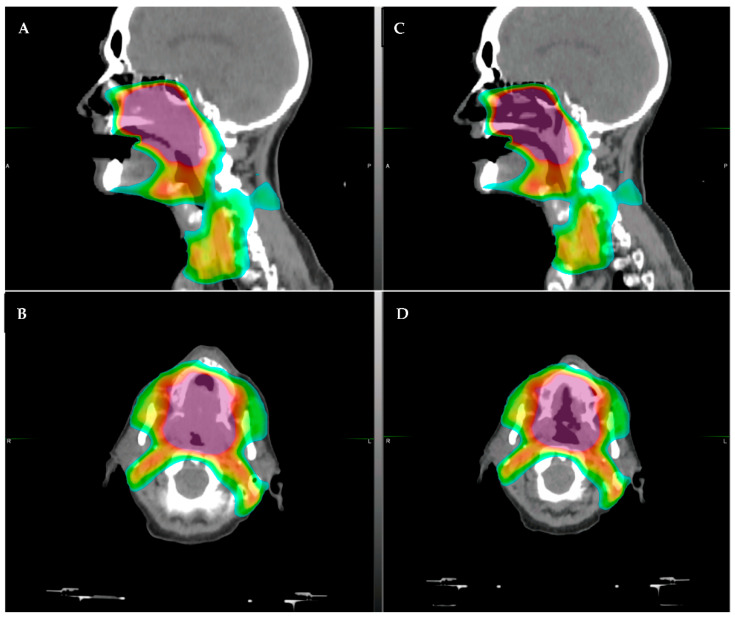
Example of a patient who had significant tumor regression after only 30 Gy of radiotherapy (of planned 70 Gy course). The patient had a large soft palate primary with extension into the nasal cavity. (**A**,**B**): original CT simulation, sagittal (**A**) and axial (**B**), respectively. (**C**,**D**): repeat CT simulation after approximately 36 Gy showing significant tumor response in the area of high dose, (**C**) sagittal and (**D**) axial. The color shading represents the radiotherapy dose distribution, with the purple/red representing areas of highest dose.

Integration of CBCT into RT has also allowed for better visualization of changes seen during the course of treatment for HNSCC, which typically lasts 6–7 weeks. Geometric plan adaptation in HNSCC is typically driven by large tumor response or large anatomic shifts due to weight loss and is important due to unintended dosimetric changes that may occur during the course of treatment that may cause unintended toxicities or affect tumor control during the course of treatment [5] (example of patient with significant tumor regression in Figure 1). Early studies assessing adaptation driven by changes seen on CBCT suggest that this approach is feasible and efficacious [6]. However, the structures that define modern approaches to RT (salivary glands, pharyngeal constrictors, lymph nodes) remain poorly visualized on CBCTs. CBCTs are limited in their ability to differentiate the varying soft tissues relevant to HNSCC.

Magnetic resonance imaging (MRI) has become widely used clinically in HNSCC. MRIs allow for better soft tissue delineation and are of particular use in visualizing perineural invasion, extracapsular extension [7,8], and muscle invasion, all of which have importance in the diagnosis and treatment of HNSCC. Integrating MRI into IGRT (MRgRT) offers an opportunity to utilize these features and allow for further improvements in RT precision. Importantly, better integration of MRI into IGRT may also expand the role of adaptive RT [9]. Early studies assessing the feasibility of off-line adaptation using MRgRT suggest that this approach is efficacious [10] and may allow for improvements in radiotherapy planning.

Early attempts to integrate MRI into IGRT was hampered by the effect of the magnetic field used to create proton spin on the secondary electrons generated by RT [11,12]. However, recent advances in technology allowed for the development of an MRgRT linear accelerator. Currently, two machines are Food and Drug Administration (FDA) approved and commercially available—one with a 0.35 Tesla (T) MRI (ViewRay MRIdian) and one with a 1.5 T MRI (Elekta Unity). These machines show promise in improving IGRT, with their better delineation of soft tissue relative to CBCT; however, significant work is needed to further the clinical use of MRgRT for HNSCC. This review describes the current use of MR-Linac, delineates the challenges in use, and proposes future research directions in HNSCC.

## 2. Current Use of MR-Linac in HNSCC

MRgRT is currently in limited use clinically, with only approximately 150 machines out in clinical practice in the world—46 with 0.35 T and approximately 100 with 1.5 T. Publications examining the use of MRgRT are mostly in gastrointestinal and genitourinary cancers [13,14,15,16,17], where mobile organs at risk, such as bowel, substantially impact RT planning and delivery. Within the head/neck region, incorporating MRI prior to RT could potentially allow for full utilization of the imaging benefits of MRI noted above (improved soft tissue delineation, etc.). Additionally, the imaging obtained during MRgRT is obtained in the treatment position immediately prior to delivery of RT, potentially allowing for better delineation of nearby organs. Limited publications on the use of MRgRT in HNSCC exist; the current data are summarized below.

A retrospective review of the use of the first clinically implemented machine for MRgRT, an older 0.35 T Cobalt 60 machine, included treatment of 17 HNSCC patients (6%, in a study describing 316 patients) [18]. A single institution experience of 13 patients with recurrent or second primary HNSCC treated using the older 0.35 T machine with Cobalt 60 source showed effective disease control with relatively low toxicity [19]. A description of prospective treatment of 10 patients utilizing the Elekta 1.5 T system following the Radiotherapy predicate studies, Idea, Development, Exploration, Assessment, Long-term evaluation conceptual framework for technical development (R-IDEAL) [20] showed that use of adaptive MRgRT is safe and feasible for HNSCC [21]. Prospective treatment with MRgRT within the multi-institutional MR-Linac Consortium on the MOMENTUM study (NCT04075305) evaluating use of the 1.5 T system included treatment of 13 patients with HNSCC and showed good tolerability of the MRgRT approach [22]. A single-institution registry study of 18 patients treated with MRgRT showed that the clinical outcomes were similar to standard RT approaches [23]. Thus, the existing limited data suggest that the use of MRgRT to adapt treatment for HNSCC is safe and feasible; however, it remains unknown whether MRgRT offers improved outcomes without compromising tumor control as compared with standard IGRT.

There are multiple ongoing or planned trials integrating MRgRT into treatment of HNSCC. The MR-ADAPTOR trial (NCT03224000) led by MD Anderson is an ongoing randomized trial utilizing the 1.5 T system, comparing standard treatment to MRI-adapted treatment in patients with human papillomavirus-related oropharyngeal squamous cell carcinoma [24]. The MARTHA trial in Switzerland (NCT03972072) aims to assess the feasibility of reducing xerostomia using the 0.35 T system. The INSIGHT-2 trial in the United Kingdom (NCT04242459) aims to personalize HNSCC dose using MRgRT. A Canadian trial is planned to assess the potential of implementing stereotactic body RT in HNSCC (NCT04809792) utilizing the 1.5 T system. Thus, while there is currently a paucity of data in utilization of MRgRT for treatment of HNSCC, multiple ongoing trials are aiming to further the use of this system and provide additional information. However, challenges to wider implementation in the treatment of HNSCC exist.

## 3. Challenges with MR-Linac in HNSCC

**Figure 2:**
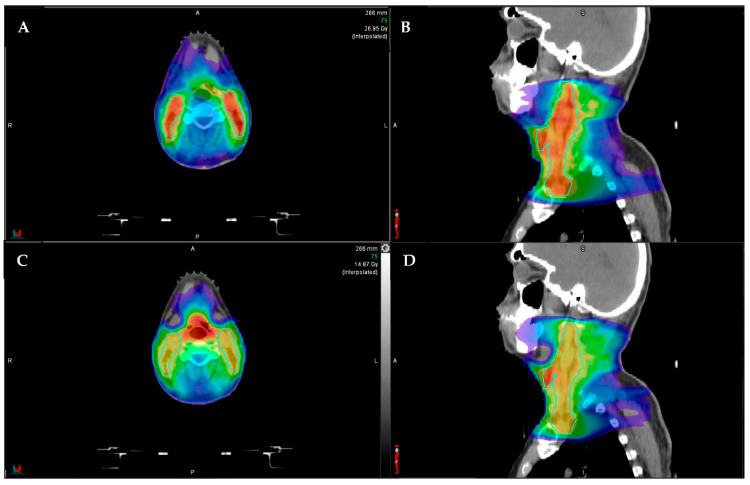
Example comparison between step-and-shoot intensity modulated radiotherapy (IMRT; (**A**) axial, (**B**) sagittal) and volumetric modulated arc therapy (VMAT; (**C**) axial, (**D**) sagittal). The color shading represents the radiotherapy dose distribution, with the red representing areas of highest dose. The aqua outlined structures represent the elective lymph node coverage. Note that the VMAT plan (**C**,**D**) has better sparing of the submandibular salivary glands (anterolateral structures), and the IMRT plan has higher hotspots ((**A**,**B**), more red color in the outlined lymph node volume).

The technical requirements for integration of an MRI into a linear accelerator used for treatment with RT required novel techniques to integrate the magnet and radiation-producing components of the machine. The Lorentz forces from the magnetic field are detrimental to moving charged particles emitted from the electron gun, and radiofrequency emissions from linear accelerators can degrade magnetic fields. In order to compensate for these constraints, the linear accelerator is housed inside a set of two shields. The shielding adds weight and complexity to the gantry, and thus the gantry cannot rotate while the radiation beam is on. This technical requirement limits the machine to a step-and-shoot intensity-modulated approach to RT and precludes the use of volumetric modulated arc tomography (VMAT). VMAT is now standardly used in the treatment of HNSCC and offers an optimal method for the sparing of critical nearby tissues in treatment of HNSCC with faster treatment time [25]. An example of VMAT versus a step-and-shoot intensity-modulated approach is provided in Figure 2. The inability of utilizing VMAT represents a significant barrier to the implementation MRgRT in treatment of HNSCC. Further research and technical development of MRgRT to allow the delivery of VMAT would offer more options for efficient HNSCC treatments.

The main advantage of integrating an MRI is the image quality and soft tissue delineation. High-quality 3 T magnets are standardly implemented in clinical use. However, the machines currently commercially available offer a 1.5 T and 0.35 T magnet. While the 1.5 T magnet may offer better imaging, the 0.35 T system offers higher frames per second, faster real time tissue tracking, automated gating, lower electron return effect, better patient set, and lower interleaf leakage [13,26,27,28,29,30,31]. Thus, the significant clinical benefits available with the lower strength magnet have made its clinical implementation attractive.

Specifically related to the use of the 0.35 T magnet in HNSCC, it is unclear whether the level of detail available on the machine will allow for sufficient soft tissue delineation to have optimal impact in HNSCC. Additional research into identifying and developing optimal sequences specific to HNSCC, optimizing coil design for imaging of the head/neck region, and determining ability to assess physiologic changes specific to RT are needed.

Finally, treatment of HNSCC with modern techniques requires a robust setup and typically includes the use of an immobilization device such as an Aquaplast mask covering the face and shoulders to minimize changes in positioning day-to-day. Additionally, custom devices such as a bite block to depress or move the tongue, dental guards to minimize backscatter, or bolus to increase the dose to the skin are often added to aid in setup for treatment of HNSCC. Current approaches to MRI-safe setup options include thermoplastic masks (CIVCO Medical Solutions, Coralville, IA, USA), setup with vacuum bags, or custom devices. Identifying robust MRI-compatible ways to immobilize patients is needed. The need for rigorous immobilization may be mitigated by the improved imaging obtained using MRI. Integration of an MRI-compatible immobilization device with MRI coils and other custom devices used during setup requires further work and research.

In summary, technical challenges to the implementation of MRgRT for HNSCC include inability to deliver VMAT, the use of lower-strength magnets with differing image qualities, and the need for further evaluation of immobilization techniques. Additional research in these areas is warranted.

## 4. Developing Areas

MRgRT offers the opportunity to obtain regular MRIs during the course of radiotherapy and may allow for both reactive changes in RT planning based on anatomical changes and potentially preventative changes in RT planning based on functional or physiologic changes seen on daily high-contrast imaging.

**Figure 3:**
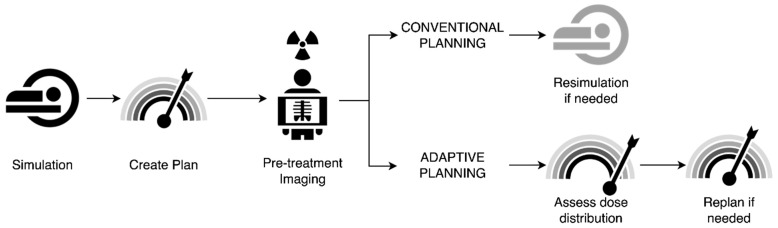
Example workflow of conventional versus adaptive radiotherapy planning. In conventional planning, patients undergo simulation, with creation of a radiotherapy plan that is delivered with standard pre-treatment imaging. If anatomic shifts are noted on pre-treatment imaging, resimulation is performed with replanning. In adaptive planning, patients similarly undergo simulation with radiotherapy plan creation; however, the additional benefit of assessment of dose distribution on pre-treatment imaging allows for better assessment of whether changes in planning are needed, allowing for either on-line (at machine) or off-line replanning.

As described above, adaptive RT has long been practiced in HNSCC, where patients commonly experience tumor shrinkage or significant weight loss affecting treatment setup during treatment. Adaptive RT utilizing CT-based planning has shown promise in maintaining good efficacy of treatment and preventing excess toxicity. Adding in the use of MRIs obtained daily or weekly may provide additional information on the rate of tumor response or other anatomic changes that may impact tumor control or normal tissue toxicity. Additionally, in challenging cases such as reirradiation, better imaging of nearby critical structures and an adaptive RT approach may offer opportunities to expand the use of adaptive RT in such settings. This largely mirrors the use of MRgRT in other disease sites where plans have been adapted to account for daily changes in anatomy. Figure 3 illustrates a generic workflow for treatment and adaptation using MRgRT.

Adaptation of plans in the head/neck region could potentially be achieved in two manners. First, MRIs obtained during MRgRT that show a need for treatment plan adaptation could result in the patient’s plan being modified after the RT treatment, termed as “off-table adaptation”. Second, the patient’s plan could be directly modified while the patient is waiting to receive RT, termed as “on-table adaptation”. The latter method has been used with success in non-HNSCC treatment adaptation, with average time of adaptation being less than 30 min [32]. However, it remains unclear how this may differ in the head/neck region, and it is unclear whether on-table or off-table adaptation will offer the best outcomes for HSNCC.

One hindrance of on-table adaptation is the need to quickly delineate, or outline, the critical nearby organs at risk in a manner that is similar to physician-approved delineation while the patient is waiting to receive RT. Currently available software and approaches are based on CT images or higher-field MRI (non-MRgRT) [33]. There are no currently available autosegmentation approaches that accurately delineate important organs and targets in the head/neck region with the lower field MRI available for MRgRT, and further development of these approaches would greatly facilitate on-table adaptation. Extension of these autosegmentation approaches into standard RT planning also has the potential to improve workflow and turnaround time for RT planning in general.

Accounting for changes in tumor response by adapting RT plans based on imaging changes may even allow for personalization of RT dosing. Not all patients have the same responses to treatment: some patients experience significant tumor shrinkage and may be able to receive a lower dose of RT that may better spare a critical nearby organ; other patients have tumors that do not shrink with treatment and may benefit from higher doses of RT to better increase tumor control probability. Frequent MRI-based imaging in conjunction with RT may offer a path forward to better treatment personalization. Patients also have different motion during the course of RT; while HNSCCs have been shown to have minimal movement during a single RT treatment [34], further investigation utilizing MRgRT could allow for additional information on understanding organ motion in the head/neck area. This could potentially have implications in the treatment of laryngeal cancer. For example, the 1.5 T MRgRT system allows for 3D tracking of motion; the 0.35 T system allows for only delivering RT when the target area is in a particular 2D plane.

Additionally, the rich imaging information provided by MRIs may point to functional or physiologic information on organ function during the course of RT. Salivary gland changes seen on CBCT have been shown to predict high-grade xerostomia overdose prediction alone [35]. Early work in MRI has shown that diffusion-weighted imaging may be predictive of outcomes [36,37]. Blood volume changes seen on MRI early in the course of RT may predict oncologic outcomes [38] or even functional outcomes such as dysphagia [39]. Additional integration of clinical information such as tumor-infiltrating lymphocytes [40], genomics, or mutational status [41] may offer predictors that can be incorporated with radiomics data.

Research is needed on integrating the use of novel MRI sequences into existing MRgRT platforms, including the development of optimal imaging techniques for identifying predictive imaging biomarkers that may allow for correlation with toxicity and outcomes. Finally, the frequent imaging obtained during MRgRT provides a rich data source from which machine learning and artificial intelligence approaches [42,43] may allow for further refinement of treatment, prognostication, or personalization of treatment for patients with HNSCC. Further study of the imaging information obtained during MRgRT may point toward improvements in delivery of RT for HNSCC.

## 5. Conclusions

In conclusion, MRgRT represents a significant advance in the delivery of IGRT, marrying the clinical benefits of MRI to the delivery of RT, and this development shows promise in technically challenging disease sites such as HNSCC. Ongoing challenges to implementation include the inability of incorporating VMAT, lower image quality, and the need for further evaluation of immobilization techniques. Incorporation of further imaging research may allow the integration of novel features of MRI, including potentially utilization of MRI as an imaging biomarker or as measurement of future functional/physiologic outcomes. Further research into MRgRT may allow for individualization of radiation dosing and advancement of areas such as reirradiation, thus possibly improving outcomes for HNSCC.
